# Epidemiology and Clinical Characteristics of Congenital Hypothyroidism in an Asian Population: A Nationwide Population-Based Study

**DOI:** 10.2188/jea.JE20120113

**Published:** 2013-03-05

**Authors:** Chung-Yu Chen, Kun-Tai Lee, Charles Tzu-Chi Lee, Wen-Ter Lai, Yaw-Bin Huang

**Affiliations:** 1 School of Pharmacy, Kaohsiung Medical University, Kaohsiung, Taiwan; 2 Division of Cardiology, Department of Internal Medicine, Kaohsiung Medical University Hospital, Kaohsiung, Taiwan; 3 Department of Public Health, Kaohsiung Medical University, Kaohsiung, Taiwan; 4 Department of Internal Medicine, Kaohsiung Medical University Hospital, Kaohsiung, Taiwan; 5 Graduate Institute of Clinical Pharmacy, Kaohsiung Medical University, Kaohsiung, Taiwan; 6 Department of Pharmacy, Kaohsiung Medical University Hospital, Kaohsiung, Taiwan

**Keywords:** congenital hypothyroidism, epidemiology, early diagnosis

## Abstract

**Background:**

The incidence of congenital hypothyroidism (CH) has been increasing in Western countries, and some populations, including Asians, have a higher incidence. Delayed diagnosis and early treatment influence the outcome of CH. We investigated the incidence and clinical characteristics of CH in Taiwan.

**Methods:**

In this retrospective database study we identified cases of CH diagnosed during 1997–2008 in the Taiwan National Health Insurance Research Database (NHIRD). Patients who had a Serious Accidents and Diseases certificate were included in the incidence calculation. We focused on CH patients who were born during 1997–2003 and determined their age at diagnosis and CH-related clinical features. Mental retardation and physiological delays were evaluated with respect to age at diagnosis.

**Results:**

A total of 1482 cases were identified. Incidence during the 12-year period was 5.02 per 10 000 births. Among 1115 patients, the most common clinical features of CH were developmental delay (9.6%), constipation (11.6%), and delayed physiological development (9.1%). Congenital anomalies of the heart (7.7%), epilepsy (2.7%), and infantile cerebral palsy (3.2%) were also noted. Survival analysis showed that the risks of mental retardation (hazard ratio [HR], 3.180) and delayed physiological development (HR, 1.908) were greater when age at diagnosis was greater than 1 year.

**Conclusions:**

CH incidence was higher in Taiwan than in Western countries. Early diagnosis may decrease the risk of mental and physiological delay.

## INTRODUCTION

Congenital hypothyroidism (CH) is a common endocrine disease in newborns and affects approximately 1 in 2000 to 4000 live births. A number of epidemiologic studies have shown that CH incidence is increasing in Western countries,^1^^,^^2^ although the reasons for this trend are not clear. Multiple factors may be involved, including ethnicity, environmental factors, characteristics of birth and pregnancy, and screening programs.^3^^–^^6^ The female-to-male ratio for CH is nearly 2:1 according to reports from most screening programs.^1^^,^^5^

Delays in diagnosis and treatment of CH may cause impairment in neurological development and intelligence quotient (IQ).^7^ The symptoms and signs of CH in infants include hoarse crying, feeding problems, constipation, umbilical hernia, anterior fontanels, hypotonia, jaundice, dry skin, and hoarseness of voice.^8^ CH is the most common treatable and preventable cause of intellectual disability in children. The thyroid gland has an important role in mental and physiological development, and its absence causes developmental delay, mental retardation, and dwarfism.^9^^,^^10^ Therefore, appropriate diagnosis and treatment are important in CH. In most cases of CH, the treatment goals are ensuring normal development, growth, and intelligence. Oral levothyroxine (L-T_4_) is the most common treatment for normalizing thyroid hormone levels in patients with CH.^11^^,^^12^ A review article indicated that infants who began thyroid hormone treatment at age 12 to 30 days had an IQ 15.7 points higher than those who began treatment after age 30 days.^12^

CH incidence in Asian populations is reported to have increased more than in US populations.^1^^,^^2^ Although a number of studies have suggested that the clinical features and epidemiology of CH differ by ethnicity, data on the clinical characteristics and epidemiology of CH in Asians are relatively scarce.^4^^,^^13^^–^^15^ Moreover, a number of studies have shown that patients may have more seizures and increased risk of heart disease when receiving thyroid hormone treatment and that thyroid function differs during long-term follow-up.^16^^–^^19^ However, little is known of trends in CH incidence among Asians. Thus, medical treatment and the clinical manifestations of CH in Asians also remain to be clarified.

In Taiwan, routine neonatal screening was begun in 1984, and the neonatal screening rate is now greater than 99%. We investigated the incidence and clinical characteristics of CH in an Asian population, using a 10-year nationwide population-based database. In addition, because delayed diagnosis causes problems in mental and physical growth, we also assessed whether developmental delay (mental and physiological) varies by age at CH diagnosis.

## METHODS

### Epidemiology and data sources

In this population-based retrospective study, data on CH patients were extracted from the list of Serious Accidents and Diseases (SAD) certificates published during 1997–2008 by the Department of Health, Executive Yuan of Taiwan. CH cases were identified by using the International Classification of Disease, Ninth Revision (ICD-9) code 243. A diagnosis of CH on a SAD certificate is strictly evaluated by at least 2 pediatricians, using laboratory data from newborns (screening criteria: thyrotropin [TSH] >40 µU/ml is the standard criterion used by the Taiwan government), and all documents are rechecked by a third physician in the Department of Health before an SAD certificate is issued. Patients with SAD certificates are eligible for exemption from insurance premiums and copayments. We extracted the medical records of CH patients from the National Health Insurance Research Database (NHIRD), which includes details of outpatient, ambulatory, and hospital inpatient care, dental services, and prescription drugs. The database was provided by the National Health Research Institute (NHRI). CH patients with SAD certificates between 1997 and 2008 were included in the analysis of clinical epidemiology and medical treatment. We quote the annual numbers and birth rates from national reports published by the Ministry of the Interior (http://sowf.moi.gov.tw/stat/month/m1-02.xls). Because this study was based in part on information from the NHIRD that comprised thoroughly deidentified secondary data released to the public for research purposes, this study was exempt from full review by an ethical committee, and the informed consent of the study subjects was not necessary.

Annual CH incidence was calculated using the total number of infants in our area each year from 1997 to 2008 as the denominator, and the total number of cases (as tracked using birthdays) in each year as the numerator. Incidence is expressed as number of cases per 10 000 births and 1 case per number of births.

### Sample and age at diagnosis

To decrease selection bias arising from the time limitation of our database (the last day was defined as December 31, 2008) and from the fact that diagnosis was delayed for several years for some patients, data from patients born after 2004 might not reflect the real results of our detailed analysis. We therefore strictly focused on CH patients who were born during 1997–2003 and had at least 4 years of medical records in the NHIRD.

Age-specific clinical presentation and diagnosis were tracked back to the SAD application date. SAD certificates are evaluated by specialist physicians who have access to comprehensive laboratory data, which limits problems in diagnostic accuracy and reduces the number of false-positive cases. However, age at diagnosis was defined as the SAD certificate application date, which means that the estimates have a slight delay in this study. Age at diagnosis was divided into 3 groups: Group 1, patients younger than 3 months at diagnosis; Group 2, those aged between 3 months and 1 year at diagnosis; and Group 3, those older than 1 year at diagnosis.

### Outcome measures

CH cases were then followed up for more than 4 years to ascertain whether they had visits for long-term hypothyroidism that caused developmental delay (ICD-9-CM code 315), mental retardation (317–319), or physiological developmental delay, ie, failure to thrive (783.41), delayed milestones (783.42), or short stature (783.43).

### Statistical analysis

The following data were analyzed: sex, age at diagnosis, year of diagnosis, disease duration, diagnostic setting, medical treatment, and CH-related signs and symptoms after CH diagnosis. Several proven signs and symptoms related to CH were selected based on the ICD-9-CM codes (eTable). Medical treatment was assessed by evaluating oral levothyroxine prescriptions at outpatient visits or during hospitalization. The start of T_4_ treatment was defined as the date on which T_4_ was first prescribed. T_4_ treatment delay was defined as the period from the diagnosis date to the start of T_4_ treatment. Duration of T_4_ treatment was calculated from the start of T_4_ treatment to the date of the last prescription in our database.

We characterized CH patients in the different age-at-diagnosis groups by age, sex, diagnosis year, diagnostic setting and area, characteristics of T_4_ use, T_4_ treatment delay, duration of T_4_ treatment, and CH-related signs and symptoms during follow-up. All cases were tracked until December 31, 2008, and their diagnosis date was used in survival analysis. Each case was followed-up until the first occurrence of 1 of the outcome measures or the end of follow-up. We censored all cases that had none of the outcomes before the end of follow-up and all deaths during the follow-up period. A Cox proportional hazards model was used to assess the association of the long-term effect of hypothyroidism with age at diagnosis, sex, diagnosis year, diagnostic setting and area, characteristics of T_4_ use, T_4_ treatment delay, and duration of T_4_ treatment. Diagnosis year was categorized as 1997–2000, 2001–2003, and 2004 or later. Analyses and calculations were carried out by using SAS 9.2. All data were expressed as frequency (percentage) and mean ± SD. Data for each risk factor were assessed in unadjusted and adjusted analysis. Statistical significance was defined as a 2-sided *P* value of less than 0.05.

## RESULTS

**Figure 1.**
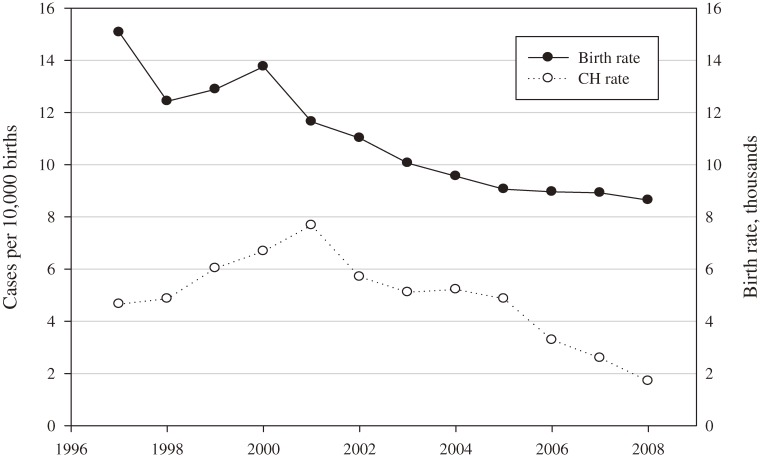
Incidence rate of CH and birth rate in Taiwan, 1997–2008.

Among 3.0 million Taiwanese infants, 1482 (708 boys and 774 girls) were identified as having CH. The 12-year average crude incidence was 5.02 infants with CH per 10 000 (Table 1). Crude incidence was 4.66 per 10 000 infants in 1997 and 1.71 per 10 000 infants in 2008 in our population. Crude incidence increased by 10% from 1997 to 2004 and decreased after 2005. Peak incidence during 1997–2008 in this population was 7.68 per 10 000 infants, in 2001. From 1997 to 2008, the female-to-male ratio for CH cases ranged from 0.79 to 1.68, and the 12-year average was 1.09. Table 1 shows that the average CH incidence per 10 000 births was 19.6% higher in girls than in boys. Although the number of CH cases fluctuated from 1997 to 2008, and the birth rate steadily decreased, there was a decreasing trend in the frequency of CH diagnoses (Figure 1).

**Table 1.** Incidence of congenital hypothyroidism^a^ in Taiwan, 1997–2008

Year	No. of Newborns			No. of CH Diagnoses				Incidence per 10 000 births			CH rate, 1 per		
			
	T	M	F	T	M	F	F/M	T	M	F	T	M	F
1997	326 002	170 047	155 955	152	67	85	1.27	4.66	3.94	5.45	2145	2538	1835
1998	271 450	141 462	129 988	132	61	71	1.16	4.86	4.31	5.46	2056	2319	1831
1999	283 661	148 042	135 619	171	85	86	1.01	6.03	5.74	6.34	1659	1742	1577
2000	305 312	159 726	145 586	203	98	105	1.06	6.68	6.14	7.21	1504	1630	1387
2001	260 354	135 596	124 758	200	105	95	0.90	7.68	7.74	7.61	1302	1291	1313
2002	247 530	129 537	117 993	141	75	66	0.88	5.70	5.79	5.59	1756	1727	1788
2003	227 070	118 984	108 086	116	49	67	1.37	5.11	4.12	6.20	1958	2428	1613
2004	216 419	113 639	102 780	113	47	66	1.40	5.22	4.14	6.42	1915	2418	1557
2005	205 854	107 378	98 476	100	56	44	0.79	4.86	5.22	4.47	2059	1917	2238
2006	204 459	106 936	97 523	67	25	42	1.68	3.28	2.34	4.31	3052	4277	2322
2007	204 414	106 898	97 516	53	22	31	1.41	2.59	2.06	3.18	3857	4859	3146
2008	198 733	103 937	94 796	34	18	16	0.89	1.71	1.73	1.69	5845	5774	5925

Total	2 951 258	1 542 182	1 409 076	1482	708	774	1.09	5.02	4.59	5.49	1991	2178	1821

T: Total; M: Male; F: Female; F/M: female-to-male ratio.

^a^Serious accidents and diseases ICD code 243.

Table 2 shows that most CH cases in Taiwan were in age group 1 (57.7%). The most frequent signs and symptoms were common cold (*n* = 645), developmental delay (*n* = 107), constipation (*n* = 129), congenital anomalies of the heart (*n* = 86), and delayed physiological development (*n* = 103). In our survey, over 95% of CH patients used oral levothyroxine, and the treatment delay was 2.78 months. Average duration of T_4_ use was nearly 4 years in our population. Half the patients were diagnosed in medical centers in northern Taiwan. In our survey, associated diseases in CH patients included mental retardation (*n* = 38), infantile cerebral palsy (*n* = 36), and epilepsy (*n* = 30). Jaundice and goiter were seen in 3.9% (*n* = 43) of cases. Speaking and voice problems were noted in 53 cases. During follow-up, only 14 patients died, and over 98% were alive at the end of follow-up.

**Table 2.** Characteristics of Taiwanese patients with congenital hypothyroidism according to age at diagnosis

Variable	Group 1 (<3 months)	Group 2 (3 months to <1 year)	Group 3 (>1 year)	Total
			
	*n* = 643	*n* = 229	*n* = 243	*n* = 1155
Age at diagnosis				
Mean (± SD), months	1.56 (0.68)	6.16 (2.39)	43.00 (22.87)	11.55 (19.88)
Diagnosis year				
1997–2000	357 (55.5)	121 (52.8)	38 (15.6)	516 (46.3)
2001–2003	275 (42.8)	99 (43.2)	70 (28.8)	444 (39.8)
2004–2008	11 (1.7)	9 (3.9)	135 (55.6)	155 (13.9)
Sex (%)				
Male	298 (46.3)	127 (55.5)	115 (47.3)	540 (48.4)
Female	345 (53.6)	102 (44.5)	128 (52.7)	575 (51.6)
Diagnostic setting (%)				
Medical center	596 (92.7)	201 (87.8)	223 (91.8)	1020 (91.5)
Metropolitan hospital	47 (7.3)	28 (12.2)	19 (7.8)	94 (8.4)
Local community hospital	0	0	0	0
Physician clinic	0	0	1 (0.4)	1 (0.1)
Diagnostic setting area (%)				
North	321 (49.9)	101 (44.3)	138 (56.8)	560 (50.2)
Central	196 (30.6)	72 (31.6)	43 (17.7)	311 (27.9)
South	119 (18.6)	50 (21.9)	57 (23.5)	226 (20.3)
East	7 (1.1)	5 (2.2)	5 (2.1)	17 (1.5)
Treatment				
T4 use (%)	617 (96.0)	217 (94.8)	226 (93.0)	1060 (95.1)
T4 treatment duration (± SD), months	52.23 (39.69)	43.5 (37.90)	38.70 (34.08)	47.49 (38.57)
Age at start of T4 use, months (± SD)	2.29 (5.31)	2.47 (6.35)	4.34 (10.72)	2.78 (7.08)
Signs and symptoms (%)				
Childhood psychoses	4 (0.6)	3 (1.3)	3 (1.2)	10 (0.9)
Development delay	53 (8.2)	33 (14.4)	21 (8.6)	107 (9.6)
Mental retardation	14 (2.2)	9 (3.9)	15 (6.2)	38 (3.4)
Infantile cerebral palsy	15 (2.3)	13 (5.7)	8 (3.3)	36 (3.2)
Epilepsy	14 (2.2)	7 (3.1)	9 (3.7)	30 (2.7)
Common cold	382 (59.4)	148 (64.6)	115 (47.3)	645 (57.9)
Inguinal hernia	6 (0.93)	4 (1.75)	1 (0.41)	11 (1.0)
Umbilical hernia	1 (0.16)	1 (0.44)	0	2 (0.2)
Constipation	69 (10.7)	36 (15.7)	24 (9.9)	129 (11.6)
Congenital anomalies of heart	46 (7.2)	23 (10.0)	17 (7.0)	86 (7.7)
Congenital anomalies of digestive system	9 (1.4)	7 (3.06)	4 (1.7)	20 (1.8)
Jaundice	25 (3.9)	9 (3.9)	9 (3.7)	43 (3.9)
Goiter	27 (4.2)	14 (6.1)	2 (0.82)	43 (3.9)
Abdominal distension	7 (1.1)	8 (3.5)	6 (2.5)	21 (1.9)
Delayed physiological development	49 (7.6)	24 (10.5)	28 (11.5)	101 (9.1)
Failure to thrive	4 (0.6)	2 (0.9)	2 (0.8)	8 (0.7)
Delayed milestones	0	1 (0.4)	0	1 (0.1)
Short stature	14 (2.2)	3 (1.3)	10 (4.1)	27 (2.4)
Speaking impairment	26 (4.0)	16 (7.0)	11 (4.5)	53 (4.8)
Hypotonia	4 (0.6)	2 (0.9)	0	6 (0.5)
Death	7 (1.1)	7 (3.1)	0	14 (1.3)

**Figure 2.**
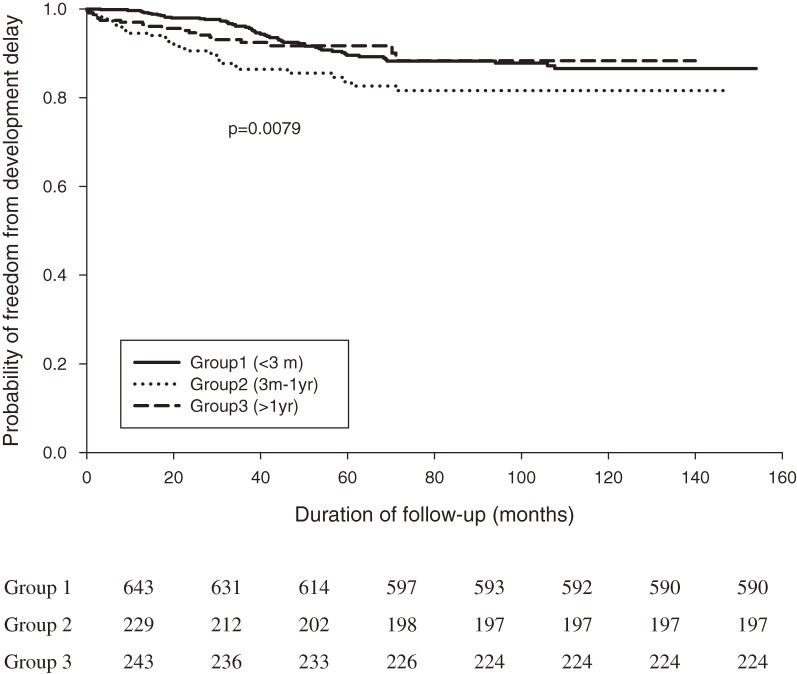
Kaplan–Meier curves for freedom from developmental delay in CH patients (*n* = 1115). Groups 1, 2, and 3 comprise patients younger than 3 months, those aged between 3 months to 1 year, and those older than 1 year at diagnosis, respectively (log-rank test, *P* = 0.0079).

**Figure 3.**
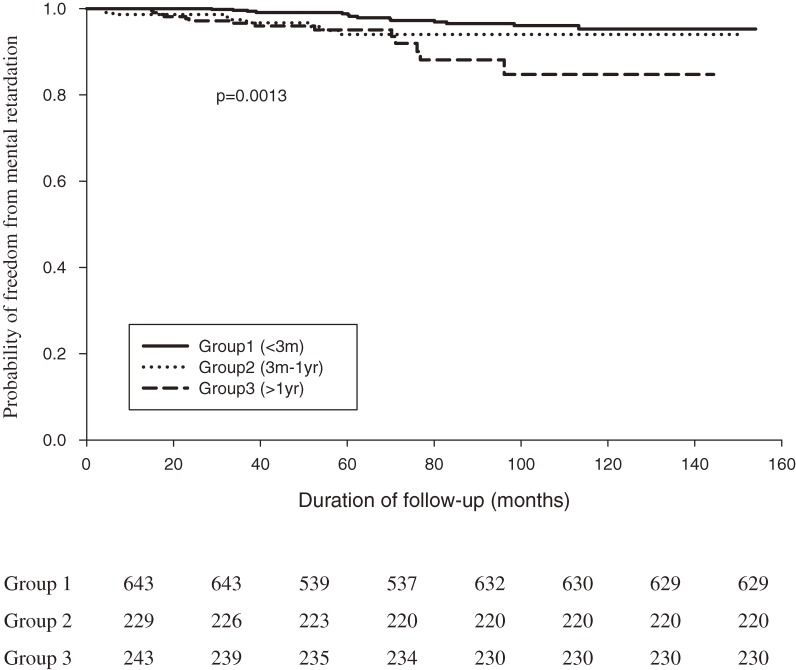
Kaplan–Meier curves for freedom from mental retardation in CH patients (*n* = 1115). Groups 1, 2, and 3 comprise patients younger than 3 months, those aged between 3 months to 1 year, and those older than 1 year at diagnosis, respectively (log-rank test, *P* = 0.0013).

**Figure 4.**
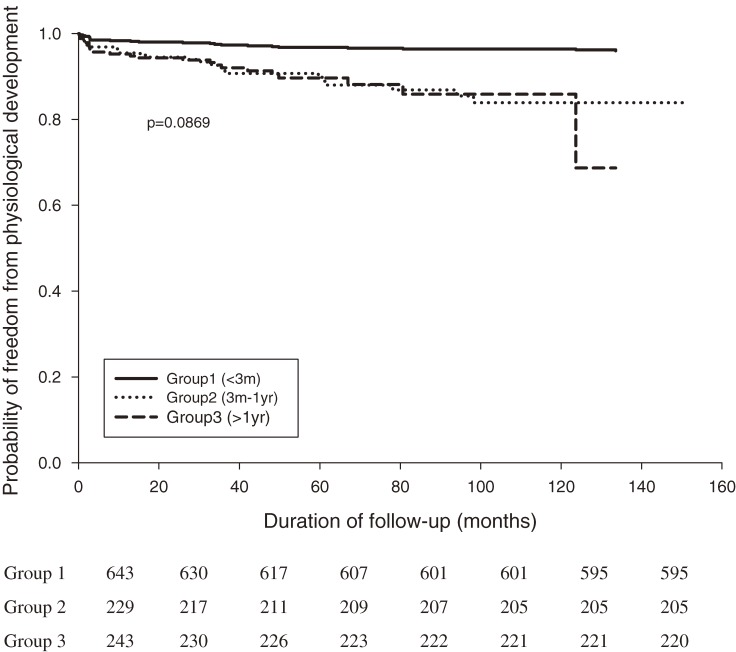
Kaplan–Meier curves for freedom from delayed physiological development (including failure to thrive, delayed milestones, and short stature) in CH patients (*n* = 1115). Groups 1, 2, and 3 comprise patients younger than 3 months, those aged between 3 months to 1 year, and those older than 1 year at diagnosis, respectively (log-rank test, *P* = 0.0869).

According to Kaplan–Meier survival curves, the incidences of developmental delay (log-rank test *P* = 0.0079) and mental retardation (log-rank test *P* = 0.0013) in CH patients significantly differed by age at diagnosis, especially among those diagnosed after age 3 months (Figures 2, 3). Moreover, the incidence of delayed physiological development (log-rank test *P* = 0.0869) was similar in the 3 age groups (Figure 4). Survival analysis (Table 3) showed that an age at diagnosis of 3 months to 1 year was associated with a higher risk of developmental delay than an age at diagnosis of less than 3 months (adjusted hazard ratio [HR], 1.957; 95% CI, 1.255–3.051). As shown in Table 4, an age at diagnosis of older than 1 year was associated with a higher risk of mental retardation than an age at diagnosis of less than 3 months (adjusted HR, 3.180; 95% CI, 1.328–7.611). As shown in Tables 3 and 4, girls had lower risks than boys of developmental delay (adjusted HR, 0.438; 95% CI, 0.291–0.660) and mental retardation (adjusted HR, 0.458; 95% CI, 0.230–0.913). Survival analysis (Table 5) showed that an age at diagnosis of older than 1 year was associated with a higher risk of delayed physiological development (ie, failure to thrive, delayed milestones, and short stature) than an age at diagnosis of less than 3 months (adjusted HR, 1.908; 95% CI, 1.083–3.362). However, the risk of delayed physiological development did not differ significantly between boys and girls (adjusted HR, 1.011; 95% CI, 0.672–1.521).

**Table 3.** Associations between independent variables and CH-related developmental delay (*n* = 1115)

Variable	Unadjusted analysis		Adjusted analysis	
	
	Hazard ratio (95% CI)	*P*-value	Hazard ratio (95% CI)	*P*-value
Age group				
Group 1	1.000		1.000	
Group 2	1.959 (1.263–3.039)	0.003	1.957 (1.255–3.051)	0.003
Group 3	1.114 (0.658–1.884)	0.688	1.121 (0.595–2.112)	0.723
Year of diagnosis				
1997–2000	1.000		1.000	
2001–2003	1.116 (0.742–1.681)	0.598	1.102 (0.704–1.726)	0.180
2004–2008	0.840 (0.423–1.667)	0.618	0.973 (0.407–2.325)	0.951
Sex				
Male	1.000		1.000	
Female	0.439 (0.294–0.656)	<0.001	0.438 (0.291–0.660)	<0.001
Diagnostic setting				
Medical center	1.000		1.000	
Metropolitan hospital	1.630 (0.912–2.915)	0.099	1.075 (0.573–2.020)	0.821
Diagnostic setting area				
North	1.000		1.000	
Central	0.485 (0.288–0.816)	0.064	0.477 (0.279–0.814)	0.007
South	0.617 (0.367–1.040)	0.070	0.604 (0.355–1.028)	0.063
East	1.092 (0.267–4.461)	0.902	1.216 (0.278–5.319)	0.795
Treatment				
T4 use	0.996 (0.246–4.042)	0.996	0.941 (0.220–4.032)	0.935
T4 treatment duration	1.002 (0.997–1.007)	0.435	1.005 (0.999–1.011)	0.119
Age at start of T4 use	0.987 (0.956–1.019)	0.432	1.000 (0.969–1.031)	0.990

**Table 4.** Associations between independent variables and CH-related mental retardation (*n* = 1115)

Variable	Unadjusted analysis		Adjusted analysis	
	
	Hazard ratio (95% CI)	*P*-value	Hazard ratio (95% CI)	*P*-value
Age group				
Group 1	1.000		1.000	
Group 2	1.992 (0.862–4.602)	0.107	1.770 (0.761–4.115)	0.185
Group 3	3.736 (1.763–7.915)	<0.001	3.180 (1.328–7.611)	0.009
Year of diagnosis				
1997–2000	1.000		1.000	
2001–2003	1.259 (0.618–2.564)	0.526	1.004 (0.454–2.221)	0.992
2004–2008	2.017 (0.706–5.759)	0.190	0.981 (0.267–3.613)	0.978
Sex				
Male	1.000		1.000	
Female	0.423 (0.215–0.831)	0.013	0.458 (0.230–0.913)	0.026
Diagnostic setting				
Medical centers	1.000		1.000	
Metropolitan hospitals	2.577 (1.132–5.868)	0.024	2.109 (0.863–5.155)	0.102
Diagnostic setting area				
North	1.000		1.000	
Central	0.678 (0.302–1.523)	0.347	0.903 (0.387–2.107)	0.813
South	0.734 (0.314–1.720)	0.477	0.780 (0.325–1.871)	0.578
Treatment				
T4 use	0.273 (0.065–1.142)	0.076	0.381 (0.076–1.915)	0.241
T4 treatment duration	0.994 (0.985–1.003)	0.202	1.000 (0.989–1.010)	0.957
Age at start of T4 use	1.009 (0.980–1.038)	0.547	1.009 (0.979–1.041)	0.549

**Table 5.** Associations between independent variables and CH-related delayed physiological development, including failure to thrive, delayed milestones, and short stature (*n* = 1115)

Variable	Unadjusted analysis		Adjusted analysis	
	
	Hazard ratio (95% CI)	*P*-value	Hazard ratio (95% CI)	*P*-value
Age group				
Group 1	1.000		1.000	
Group 2	1.520 (0.932–2.477)	0.093	1.376 (0.838–2.260)	0.207
Group 3	1.614 (0.979–2.660)	0.061	1.908 (1.083–3.362)	0.025
Diagnostic year				
1997–2000	1.000		1.000	
2001–2003	1.315 (0.859–2.013)	0.208	0.956 (0.610–1.497)	0.843
2004–2008	0.771 (0.343–1.730)	0.528	0.407 (0.158–1.046)	0.062
Sex				
Male	1.000		1.000	
Female	0.954 (0.639–1.426)	0.820	1.011 (0.672–1.521)	0.958
Diagnostic setting				
Medical centers	1.000		1.000	
Metropolitan hospitals	1.443 (0.770–2.705)	0.252	1.307 (0.643–2.656)	0.459
Diagnostic setting area				
North	1.000		1.000	
Central	0.685 (0.361–1.298)	0.246	0.748 (0.388–1.439)	0.384
South	3.526 (2.262–5.495)	<0.001	3.736 (2.363–5.908)	<0.001
East	3.463 (1.062–11.295)	0.039	3.260 (0.920–11.549)	0.067
Treatment				
T4 use	0.512 (0.162–1.625)	0.256	1.060 (0.309–3.640)	0.927
T4 treatment duration	0.996 (0.990–1.001)	0.148	0.995 (0.998–1.001)	0.090
Age at start of T4 use	0.993 (0.965–1.022)	0.654	0.978 (0.945–1.012)	0.197

## DISCUSSION

CH has been widely researched in Western countries, but the incidence of CH is considered to be higher in East Asia (Table 6).^1^^,^^3^^,^^13^^–^^15^^,^^20^^–^^24^ In the United States, the incidence of CH reportedly increased from 24.4 cases per 100 000 births in 1987 to 42.2 cases per 100 000 births in 2002.^1^ A recent report also indicated that CH incidence in the United States has increased by 3% per year.^2^ Although some research found that the increase in incidence was less marked in Quebec^25^ than in other places, an incremental increase was still noted. A review of CH incidence in California during 2001–2007 yielded an estimate of 5.02 per 10 000 infants, which was similar to that in Asian Indians (5.7 per 10 000 infants) and Chinese and Vietnamese (4.2 per 10 000 infants) but higher than that in non-Hispanic whites (3.6 per 10 000 infants) and non-Hispanic blacks (0.9 per 10 000 infants).^2^ CH incidence in East Asian countries such as Japan (6.8 per 10 000 infants)^15^ and China (4.8 per 10 000 infants)^13^^,^^14^ is higher than that in Western countries. A study of Asia-Pacific screening programs for newborns found that some Asian countries screened the entire population (Japan, China, Taiwan and Australia) and others used pilot testing or selected population screening (Pakistan and India).^4^ Therefore, the higher incidence in East Asia may be due to regional differences in screening programs for newborns. However, there have been studies on the genetics of CH in different ethnicities, as this may be an important factor in the higher incidence in East Asia.^6^ Most CH screening programs report that the female-to-male ratio is 2:1, but in the present study it was 1.09:1, which is similar to the results of a number of other studies.^26^ However, the incidence of CH in was still 19.6% higher in girls as compared with boys in our population.

**Table 6.** Epidemiologic studies of congenital hypothyroidism

Area	Country	Study period	Incidence per 10 000 births

			Crude rate
East Asia	Taiwan (present study)	1997–2008	5.0
	China^13^^,^^14^	2000–2007	4.8
	Japan^15^	1994–2002	6.8
West Asia	Turkey^22^	1991–1992	3.7
	Turkey^24^	2000–2002	4.3
	Pakistan^23^	1989–2007	1.9
Europe	Scotland^20^	1979–2003	2.3
	England^3^	1982–1997	3.4
	Italy^21^	1995–2003	4.1
North America	New York State, US^1^	1978–2005	4.8
	US (not including New York State)^1^	1987–2002	3.2

Table 1 and Figure 1 show that CH incidence increased by 10% from 1997 (4.66 per 10 000) to 2003 (5.11 per 10 000) and decreased after 2004. There are factors that can explain these trends. First, as show in Table 2, most patients who received delayed diagnoses were older than 3.5 years, but we cannot trace patient information from SADs after 2009, and the number of patients born after 2004 with a delay in diagnosis greater than 4 years would be underestimated. Second, because age at diagnosis was calculated by using the SAD certificate application date, there was a slight delay in our study. Hence, the fact that the number of confirmed cases decreased significantly from 2004 to 2008 shows that accurate diagnosis was still difficult after 2004 in our database. Third, accurate case numbers may be underestimated owing to patients who died before they could apply for an SAD certificate and to patients who did not apply for an SAD certificate. Fourth, the decrease in case numbers may be because the reduction in the birth rate in our area was faster than those in studies performed in other countries. Although Harris et al reported increasing birth rates in races with higher incidences, it is difficult to explain the present decrease in incidence.^1^ It appears that additional factors are involved in the correlation between birth rate and CH incidence. Lastly, the criterion for screening (TSH >40 µU/ml) is the standard criterion used by the Taiwan government; however, recent reports suggest that use of multiple tests (ie, T_4_ and TSH) improves detection of primary and central hypothyroidism.^27^^–^^29^ Therefore, the screening program is improving every year, and detailed screening is performed to decrease misclassification of possible newborns. However, this may increase the number of false-positive cases. On the SAD certificate, which is evaluated by 2 specialized physicians in Taiwan, the primary screening test is TSH level, and advanced screening includes TSH, free T4, T4, thyroglobulin, thyroxine-binding globulin, and thyroid sonogram or 99mTc thyroid scanning. Some screening items in the advanced screening have been used in recent years and are recommended for suspected CH patients after initial primary screening in Taiwan. Although this improves diagnostic accuracy and decreases the number of false positives, the numbers of cases may be underestimated in our database, which could affect estimates of CH incidence in Taiwan. Hence, the changing trend in crude incidence during 1997–2003 might be more accurate, and crude incidence after 2004 should be regarded as tentative.

Delayed diagnosis and treatment is a serious problem in some South Asian countries, such as Pakistan and India, where less than 30% of patients are diagnosed before age 1 year.^26^^,^^30^ Although 99% of Taiwanese infants undergo routine neonatal screening, only 78.2% of the present patients received a diagnosis before age 1 year. Padilla and Therrell^4^ studied newborn screening in the Asia-Pacific region and found that some South and Southeast Asian countries do not screen all newborns for CH. A 1997 Health Technology Assessment publication estimated the short-term costs at 18 000 GBP per year, with long-term costs rising to 174 000 GBP per year for screening 100 000 births per year.^31^ The CH burden can also be decreased when all newborns in a population are screened. CH diagnosis and treatment should be performed as soon as possible to decrease the likelihood of serious impairment of neurological development and intelligence quotient. During the last 30 years, most industrialized countries have been conducting neonatal screening programs that have decreased cerebral damage and the severity and morbidity of CH in children.^32^ Developmental delay, mental retardation, and delayed physiological development are long-term complications of hypothyroidism. A number of studies on long-term outcomes have shown that age at diagnosis, CH severity at diagnosis, and treatment adequacy have long-term effects on health-related quality of life scores and cognitive and motor outcomes.^33^^–^^35^ Our findings were consistent with studies conducted in different regions, which showed that CH patients are at greater risk of developmental delay, mental retardation, and delayed physiological development when CH is diagnosed after age 3 months. Furthermore, males had a higher risk of developmental delay and mental retardation in our study, a finding that supports research by Oerbeck et al^34^ on long-term outcomes. They found that female CH patients had better social and motor outcomes than male patients. However, a correlation between CH disease severity and progression was not observed in either sex in a study by Eugene et al.^5^ Our findings show that more than 95% of CH patients had at least 1 course of T_4_ treatment, that the duration of treatment was longer than 4 years, and that treatment started at an average age of 2 months. The lack of difference in risk remained even when patients were stratified according to duration of T_4_ treatment or the interval between diagnosis and start of T_4_ treatment. A number of studies reported that T_4_ treatment should be started within 2 weeks of a CH diagnosis.^12^ In the present report, T4 treatment delay was longer than 2 weeks, perhaps because the T4 treatment starting point was calculated from the date of first T4 prescription in NHIRD and because the estimates have a limited time delay in this study. Treatment may also have been delayed because disease severity was milder in our population. However, 1 report suggested that treatment delay was not associated with IQ or motor scores in long-term follow-up.^35^

The clinical characteristics of CH may differ between Asian and Western countries. There are few studies of the signs and symptoms of CH-associated complications in Asian patients. Our study confirmed that our population also experienced the common signs and symptoms, ie, developmental delay, mental retardation, constipation, and delayed physiological development (ie, failure to thrive, delayed milestones, and short stature). The frequency of congenital anomalies of the heart was 7.7% in our patients. Wu et al found that the prevalence of congenital heart defects (CHD) in Taiwanese newborns was 1.3%^36^ and that prevalence was higher among CH patients than in the general population of newborns. Similarly, recent clinical studies also showed that CHD patients have a 10-fold higher risk of CH than do general newborns.^37^ The correlation between CHD and CH may be due to embryonic development between the thyroid gland and heart, and the great vessels. Results showing a higher prevalence of CHD in CH patients suggest that CH patients should be evaluated by echocardiography and monitored carefully.

Epilepsy has also been discussed in thyroid-related disease and treatment. Thyroid hormones have an important role in perinatal development of the central nervous system, and recent studies have reported that the incidence of seizure in people with hyperthyroidism is higher than in individuals with normal hormone levels.^17^ Our data show that the frequency of seizure in CH patients was 2.7%. Although no study has shown a correlation between seizure and CH in newborns or children, a recent case report described an infant suffering from intractable neonatal seizures with CH.^38^ More evidence is needed to determine the exact cause and mechanism. It is important to note that infantile cerebral palsy occurred in 3.2% of patients in our survey. There are limited data on the correlation between CH and infantile cerebral palsy, but it may be prudent to monitor CH patients for signs and symptoms of infantile cerebral palsy after CH diagnosis.

This study had a larger sample and a longer study period than any other analysis of the epidemiology and clinical characteristics of CH in Taiwan. Although a number of studies have been performed in Asia, information on CH epidemiology are lacking in this population. We conducted this study using a nationwide database that had the complete medical history for each patient. This database was obtained from the single-payer National Health Insurance program, which enrolled 98% of Taiwan’s population. In our study, we identified CH patients from SAD certificates and clinical coding, but CH cases need to be evaluated by laboratory data and image testing to ensure that their SAS certificate is accurate. The database had few problems with regard to diagnostic accuracy. However, there are some limitations in the present study. First, the incidence for the SAD certificates was calculated using the application date, so the estimates have some time delay. The number of cases might have been underestimated if infants died before an SAD certificate could be issued. Unfortunately, the database used in our study was a double-identified, secondary, non-random sample database. Thus, it is difficult to determine if underestimation occurred. Second, infant characteristics such as body weight, area of residence, maternal and paternal characteristics, health-care policy, socioeconomic status, and disease severity were not available from the NHIRD. A number of reports indicate that CH severity influences treatment efficacy and disease progression. In the present study, we were unable to evaluate CH severity and compare the severity of associated signs and symptoms with data from other countries. Third, we had no imaging findings or data on hormone levels for CH patients, and these might be potential confounders. Fourth, we used data on hospital and outpatient visit claims to measure medical treatment and duration. Some patients and families may not have completely adhered to the instructions of physicians, which could lead to errors in the measurement of exposure and duration. Fifth, it is difficult to calculate T_4_ starting dose or continuation dose from the database, and these might be potential confounders.

### Conclusions

In summary, the incidence of CH in Taiwan remains higher than in Western countries. However, the marked increase in incidence in Taiwan was not significant during 1997–2008. The epidemiology of CH appears to be similar to that in some other Asian countries but not to that in Western countries. The signs and symptoms of CH are similar to those reported in Western countries, ie, constipation, jaundice, intellectual disability, developmental delay, and delayed physiological development. Congenital anomalies of the heart, epilepsy, and infantile cerebral palsy also occur in some CH patients. More surveillance of CH-related diseases needs to be conducted in the future. Data from CH patient surveys show that over 78% of CH patients are diagnosed before age 1 year and that early diagnosis can decrease the risks of developmental delay, mental retardation, and delayed physiological development.

## ONLINE ONLY MATERIALS

eTables. The World Health Organization (WHO) published International Classification of Disease, Ninth Revision (ICD-9) code numbers used for present analysis.
