# Supplementary material for: Epidemiology and Clinical Characteristics of Congenital Hypothyroidism in an Asian Population: A Nationwide Population-Based Study
Source: J Epidemiol. 2013 Mar 5;23(2):85–94. doi: 10.2188/jea.JE20120113 (PMC3700243; doi:10.2188/jea.JE20120113)
Supplement: eTables. — The World Health Organization (WHO) published International Classification of Disease, Ninth Revision (ICD-9) code numbers used for present analysis. [file je-23-085-s001.pdf]

eTable. The World Health Organization (WHO) published International Classification of Disease, Ninth Revision (ICD-9) code numbers used for present analysis

Disease Category	ICD-9-CM Number
Childhood psychoses	299
Development delay	315
Mental retardation	317-319
Infantile cerebral palsy	343
Epilepsy	345
Common cold	460,465-466
Inguinal hernia	550
Umbilical hernia	551.1,552.1,553.1
Constipation	564
Congenital anomalies of heart	745-746
Congenital anomalies of digestive system	751
Jaundice	774,7824
Goiter	240.9
Abdominal distension	7873
Delayed physiological development	783.4
Failure to thrive	783.41
Delayed milestones	783.42
Short stature	783.43
Speaking impairment	784
Hypotonia	781.3
